# Effects of Dietary Phytol Supplementation on Growth Performance, Immunological Parameters, Intestinal Bacteria, and Prevention of Oxidative Stress Following Transportation of Nile Tilapia, *Oreochromis niloticus*

**DOI:** 10.1155/2024/7039179

**Published:** 2024-02-07

**Authors:** Saman Ahani, Sara Ahani, Morteza Yousefi, Ali Taheri Mirghaed, Afaf N. Abdel Rahman

**Affiliations:** ^1^Science and Research, Islamic Azad University, Tehran, Iran; ^2^Department of Veterinary Medicine, RUDN University, Miklukho-Maklaya St, Moscow 117198, Russia; ^3^Department of Aquatic Animal Health, Faculty of Veterinary Medicine, University of Tehran, Tehran, Iran; ^4^Department of Aquatic Animal Medicine, Faculty of Veterinary Medicine, Zagazig University, P.O. Box 44511, Zagazig, Egypt

## Abstract

Nile tilapia, *Oreochromis niloticus*, (2.00 ± 0.02 g) were reared in 16 70-L tanks (40 individual/tank) and fed diets (approx. 345 g/kg protein, approx. 87 g/kg crude fat) containing 0 (CTL), 75 (PH-75), 150 (PH-150), and 300 (PH-300) mg/kg phytol (*n* = 4). After 60 days of feeding (4% daily), growth performance, humoral immune parameters, and gut bacteria were analyzed. Also, hepatic antioxidant parameters were determined before and after the fish were transported in plastic bags for 6 hr. The results showed that PH-75 exhibited the highest final weight (*P* < 0.001), weight gain (*P* < 0.001), feed intake (*P* < 0.001), feed efficiency (*P*=0.015), plasma lysozyme activity (*P*=0.004), and intestinal *Lactobacillus* sp. population (*P*=0.017), among the treatments. The highest plasma alternative complement activity (*P*=0.006) and the lowest intestinal total viable bacteria (*P*=0.027) were observed in PH-75 and PH-150. The highest plasma alkaline phosphatase activities were observed in PH-75 and PH-300 (*P*=0.014). The highest blood leukocyte (*P*=0.008), monocyte (*P*=0.010), and eosinophil (*P* < 0.001) were observed in PH-300, while the highest blood neutrophil was observed in all phytol treatments (*P* < 0.001). The highest hepatic lipid peroxidation was observed in PH-300, whereas PH-75 and PH-150 showed the lowest values (*P* < 0.001). The highest hepatic reduced glutathione was observed in PH-75, also PH-150 exhibited significant elevation in this parameter, compared to CTL (*P* < 0.001). Transportation led to significant elevations in the hepatic antioxidant enzymes' activities in CTL, PH-75, and PH-150; the highest activities were related to PH-75 and PH-150 treatments, which had also the highest post-transportation survivals (*P* < 0.001). In conclusion, phytol is a suitable feed supplement for Nile tilapia, improving growth performance and welfare, particularly at 75 mg/kg.

## 1. Introduction

The Nile tilapia, *Oreochromis niloticus*, which has a global production of 4.44 million tons in 2019, is the most widely cultivated tilapia species [1]. Nevertheless, the industry of Nile tilapia aquaculture is vulnerable to disease outbreaks, making it imperative to enhance fish health and prevent such occurrences [2]. Furthermore, global regulatory bodies have prohibited the use of chemical drugs and antibiotics in aquaculture [3]; thereby, obliging fish farmers to focus on enhancing fish health and preventing disease outbreaks by using functional diets, among other managerial strategies [4].

Studies have demonstrated that herbal feed additives have various beneficial properties, including promoting growth, stimulating the immune system, acting as antioxidants, and tackling microbial infections. It has been found that these additives improve the overall health and immune responses of host organisms. They have also been shown to be effective in stress management in aquaculture [5–7]. Stress is a common problem in farmed fish, which can disrupt their physiological balance and affect their growth, immune system, and health of various organs [8]. The transportation of fish, a common practice in aquaculture, can exacerbate these stress-related problems and lead to oxidative stress and immunosuppression [9–11]. However, studies have shown that certain herbal supplements, such as turmeric, *Curcuma longa* [10], and aloe vera, *Aloe vera* [12], can mitigate these negative effects. Despite this, there is still a need for further research on new herbal additives that can mitigate the disadvantages of transportation in fish.

The phenolic compounds found in plants have beneficial properties that make them suitable for inclusion in fish feed. Anti-inflammatory, antimicrobial, and antioxidant activities of these compounds can mitigate the negative effects of stress [13]. Synthetic forms of these compounds, known as nature identical compounds (NICs), offer advantages over natural compounds. Since NICs are more readily available and more cost-effective to produce, they are a practical choice for the aquaculture industry [13].

Phytol is a NIC with immune boosting, antibacterial, and antiviral properties. The production of phytol is widespread, as it serves as a substrate for the production of vitamins E and K [14]. The benefits of phytol in fish have only been examined in a few studies. For example, goldfish, *Carassius auratus*, fed with a feed containing phytol showed resistance to bacterial infection and improvement in hematological parameters after infection [15]. The antioxidant, antistress, and hepatoprotective properties of phytol helped mitigate the toxic effects of water ammonia on common carp, *Cyprinus carpio*, by acting as a hepatoprotective agent [16]. These results suggest that phytol may be a promising feed additive in aquaculture, but further investigation is needed to understand its physiological functions in other fish species.

Therefore, this study sought to evaluate the effects of dietary phytol incorporation on the growth performance, immunological parameters, post-transportation survival, and antioxidant responses of Nile tilapia.

## 2. Materials and Methods

### 2.1. Diets

Diets of similar macronutrient compositions were formulated using Windows User-Friendly Feed Formulation (WUFFDA-v.1). The only difference in diet was the amount of phytol added at the expense of bentonite, and four levels of phytol (0 (CTL), 75 (PH-75), 150 (PH-150), and 300 (PH-300) mg/kg) were tested (Table 1). Before addition to the other ingredients, phytol was mixed with the dietary oils. The ingredients were mixed for 20 min and moisturized to form a dough, which was then processed into pellets using a meat grinder. The pellets were dried overnight and stored at 4°C until use. The proximate compositions of the diets were determined according to AOAC [17]. The crude protein was determined using the Kjeldahl method after it was digested in sulfuric acid and the resulting ammonia was bound with boric acid. The solution was then titrated and the amount of nitrogen was determined and multiplied by 6.25 to calculate the crude protein content. Crude fat was determined as an ether extract using petroleum ether as a solvent in a Soxhlet apparatus (Gerhardt, Germany). Crude ash was measured using an electric furnace (550°C; 8 hr) by burning a 1-g sample and subtracting the weight of the sample before and after burning. Crude fiber was measured by placing a 1-g sample in a filter bag, boiling in 0.1 M NaOH, and then boiling in 0.1 M HCl. The crude fiber content was calculated by subtracting the weights of the samples before and after digestion. The moisture content of the samples was determined in the oven (105°C; 24 hr) by subtracting the weights of the samples before and after drying.

### 2.2. Feeding Trial

Eight hundred Nile tilapia (∼1.5 g) were purchased from a local farm (Kashan, Iran) and brought to the laboratory where they were housed in a 1,000-L tank. The fish were acclimatized to the conditions for 1 week by feeding them with CTL diet. They were then sorted to exclude individuals with outlier sizes (10% of the highest and lowest sizes). The remaining 640 fish were housed in 16 70-L tanks (58 cm × 30 cm × 40 cm) with 40 fish per tank. The fish within each tank were bulk-weighed both prior to stocking (total biomass of 77.2–83.2 g/per tank) and biweekly after that (to adjust the feed amounts). The fish were daily fed equal to 4% of biomass, divided into three meals (1.4% at 7:00, 1.4% at 13:00, and 1.2% at 18:00). No feed wastes were detected 20 min after each meal.

The tanks were thoroughly cleaned on a weekly basis and regularly aerated. The water quality was monitored using digital instruments, including a temperature of 27.3 ± 0.73°C, dissolved oxygen of 6.60 ± 0.76 mg/L, pH of 7.4 ± 0.53, and total ammonia of 0.85 ± 0.09 mg/L. The photoperiod was adjusted to a 12-hr light/dark cycle by means of lamps, and the fish were reared in these conditions for a duration of 60 days.

### 2.3. Growth Parameters

The growth and feed efficiency parameters were determined in all treatments according to the following equations:

(1) $$\begin{array}{c} \text{Weight gain (\%)}=100\times \left[\frac{\text{Final weight}-\text{Initial weight}}{\text{Initial weight}}\right], \end{array}$$

(2) $$\begin{array}{c} \text{Feed efficiency (\%)}=100\times \left[\frac{\text{Final weight}-\text{Initial weight}}{\text{Feed intake}}\right], \end{array}$$

(3) $$\begin{array}{c} \text{Specific growth rate (SGR; \%/day)}=100\times \left[\frac{{\text{ln}}_{\text{Finalweight}}-{\text{ln}}_{\text{Initialweight}}}{60}\right]. \end{array}$$

### 2.4. Pretransportation Sampling and Processing

After 60 days, three fish per tank were gently caught by a dip net, anesthetized in a eugenol bath (50 *µ*L/L), and their blood samples were taken for counting the blood leukocytes (WBC) and assessing plasma immunological parameters. The blood samples were collected by caudal puncture using heparinized syringes and collected in 2-mL plastic tubes. A portion of the blood samples were used for hematological assay and the other portion was centrifuged (4°C for 7 min; 5,000x *g*) for plasma separation, which was kept at −70°C until analysis. The fish were then euthanized by a sharp blow to the head, and their weights were recorded. To assay antioxidant parameters, the fish abdominal cavities were opened and a piece of the liver was dissected, washed with distilled water, frozen in liquid nitrogen, and stored at −70°C until analysis. A piece of the posterior intestine was then dissected, washed with sterile physiological saline, and immediately used for microbial culture. One additional batch of three fish per tank was caught, anesthetized, and then used for indirect skin mucus collection. The fish were individually placed in separate plastic bags containing 5 mL of Tris-buffered saline, comprising 50 mM Tris-HCl, 150 mM NaCl, pH 8.0 [18], and subsequently rubbing for a brief period. The combination of the mucus and buffer was collected and centrifuged at 4°C for a duration of 15 min (13,000x *g*). The supernatants were collected and stored at a temperature of −70°C until the immunological analysis.

### 2.5. Analysis

#### 2.5.1. Hematological Assays

Fresh blood samples were used for WBC counting, using the Dacie diluting solution according to Dacie and Lewis [19]. Differential WBC count was performed after preparation of the blood smear and staining by Giemsa, following Blaxhall [20].

#### 2.5.2. Plasma Immunological Assays

The evaluation of plasma lysozyme activity was conducted by analyzing the lysis rate of *Micrococcus luteus*, as per Ellis [21]. A phosphate buffer pH 6.2 was used as the reaction medium. Thirty microliters plasma was mixed with 1 mL of the bacterial suspension and decreases in optical density was recorded for 5 min at 550 nm. Each 0.001 decrease in the optical density per minute was deemed as one unit of lysozyme activity. The plasma alkaline phosphatase (ALP) activity was determined using a commercial kit (Man Co., Tehran, Iran) as suggested by Esmaeili et al. [22]. This kit works by hydrolysis of p-nitrophenylphosphate (substrate) and production of p-nitrophenol with a yellow coloration. During 3 min, the optical density was measured at 405 nm and the enzyme's activity was calculated using a standard solution furnished by the manufacturer.

The activity of plasma alternative complement (ACH50) was determined by measuring hemolytic activity against sheep erythrocytes. The reaction medium was a barbital buffer (pH 7.0) containing ethylene glycol-bis(-aminoethyl ether)-N, N,N′, N′-tetraacetic acid, magnesium, and gelatin. A defibrinated blood of sheep, obtained from Darvash Co., Tehran, Iran, underwent a three-step process of washing with the barbital buffer, followed by the suspension of 50 *µ*L of the packed cells in 950 *µ*L of the same buffer. In a round-bottom tube, 50 *µ*L of the sheep erythrocyte suspension, 50 *µ*L of the samples, and 50 *µ*L of the buffer were added and mixed gently. After 90 min of incubation at room temperature, the hemolytic activity was stopped by adding a barbital buffer containing ethylenediaminetetraacetic acid. After a period of 5 min of centrifugation, the hemolysis of the tubes was determined at 420 nm, and the amount of sample producing 50% hemolysis was quantified through a log–log plot. The ACH50 activity was calculated according to Yano [23].

#### 2.5.3. Skin Mucosal Immunological Assays

The skin mucosal lysozyme and ALP activities were determined as described for the plasma samples. The skin mucosal peroxidase activity was determined using 3,3′, 5,5′-tetramethylbenzidine hydrochloride (TMB), as the substrate, and hydrogen peroxide. The dissolved TMB was dissolved in dimethylsulfoxide to obtain a concentration of 41 mM. To prepare a working solution of TMB, 200 *µ*L of this solution was added to 8 mL of acetate buffer (205 mM, pH 4.0). Afterward, 500 *µ*L of the TMB working solution, 250 *µ*L of the samples, and 340 *µ*L of hydrogen peroxide (30%) were mixed and left at 40°C for 10 min. Subsequently, 1.2 mL of sulfuric acid 0.2 M was incorporated into the mixture to halt the reaction. After centrifugation (9000x *g* for 4 min), the absorbance of the supernatant was measured at 450 nm. The absorbance of the samples at the time zero was obtained by incorporating sulfuric acid at the time zero. The photooxidation of TMB was estimated using a control sample [24].

The soluble protein concentrations of the mucus homogenates were measured using the pyrogallol red method, using a commercial kit from Zist Chem Co., Tehran, Iran.

#### 2.5.4. Hepatic Antioxidant Parameters' Assays

The determination of hepatic antioxidant parameters was conducted using commercial kits supplied by Zellbio Co. (Deutschland, Germany). The concentration of glutathione (GSH) was determined by measuring its reaction with 5,5′-dithiobis-(2-nitrobenzoic acid) at 412 nm. The glutathione peroxidase (GPx) assay involved the addition of GSH to the samples and subsequent conversion into oxidized glutathione (GSSG) by the GPx enzyme present in the samples. It is then converted back to GSH by glutathione reductase activity, which requires nicotinamide adenine dinucleotide phosphate (NADPH). The decrease in NADPH concentration is directly proportional to the activity of GPx, as measured at 340 nm. The identical procedure is followed for determining GR activity, with the exception of incorporating GSSG into the samples in lieu of GSH. The content of thiobarbituric acid reactive substances (TBARS) was determined based on the reaction with thiobarbituric acid at 95°C, after deproteinization with trichloroacetic acid and in the presence of butylated hydroxytoluene. The pink coloration intensity was proportional to the TBARS concentration and was measured at 550 nm.

The soluble protein concentrations of the liver homogenates were measured using the pyrogallol red method, using a commercial kit from Zist Chem Co., Tehran, Iran.

#### 2.5.5. Intestinal Total Viable Bacteria (TVB) and Lactic Acid Bacteria (LAB) Counting

Subsequent to the sampling, the intestinal samples were promptly homogenized in a sterile porcelain mortar. After the preparation of the homogenate, a variety of dilutions spanning the range of 10^−1^−10^−7^ were prepared by utilizing 0.9% physiological saline. A volume of 0.1 mL was extracted from the desired dilutions under sterile conditions and transferred to a plate containing nutrient agar and De Man–Rogosa–Sharpe media for the purpose of counting TVB and LAB, respectively. After 72 hr of incubation at room temperature under aerobic conditions, the colony-forming units (CFU) were counted [25].

### 2.6. Fish Transportation and Post-Transportation Sampling

Upon completion of the pretransportation sampling, the fish were subjected to 6-hr transportation in 60-L plastic bags. Each bag consisted of 4–5 L of water and 30 fish, which were placed to resemble a fish stocking density of 100 g/L. The bags were then filled with pure oxygen (two folds of water volume), tight sealed, and transported in a van for 6 hr. At the destination, three fish per bag were netted, anesthetized with eugenol (50 *µ*L/L), and euthanized by a sharp blow on the head. Subsequently, the weights of the fish were recorded, their abdominal cavities were opened, and a portion of the liver was dissected. The samples were washed with distilled water and frozen in liquid nitrogen for antioxidant parameters assays. The sampling and antioxidant analysis were carried out similar to that of before fish transportation (see above). In order to record their mortality, the remaining fish were bulk-weighed and returned to their respective tanks.

### 2.7. Statistical Analysis

The normal distribution of the data was determined using the Shapiro–Wilk test. The analysis of growth performance, immunological parameters, intestinal bacteria, and survivals was conducted through one-way analysis of variance (ANOVA) and Duncan tests. The analysis of hepatic antioxidant parameters was conducted through a two-way repeated-measure ANOVA and the Duncan test. All analyses were performed using the Statistical Package for the Social Sciences (SPSS) version 22 with a significance level of 0.05.

## 3. Results

**Figure 1:**
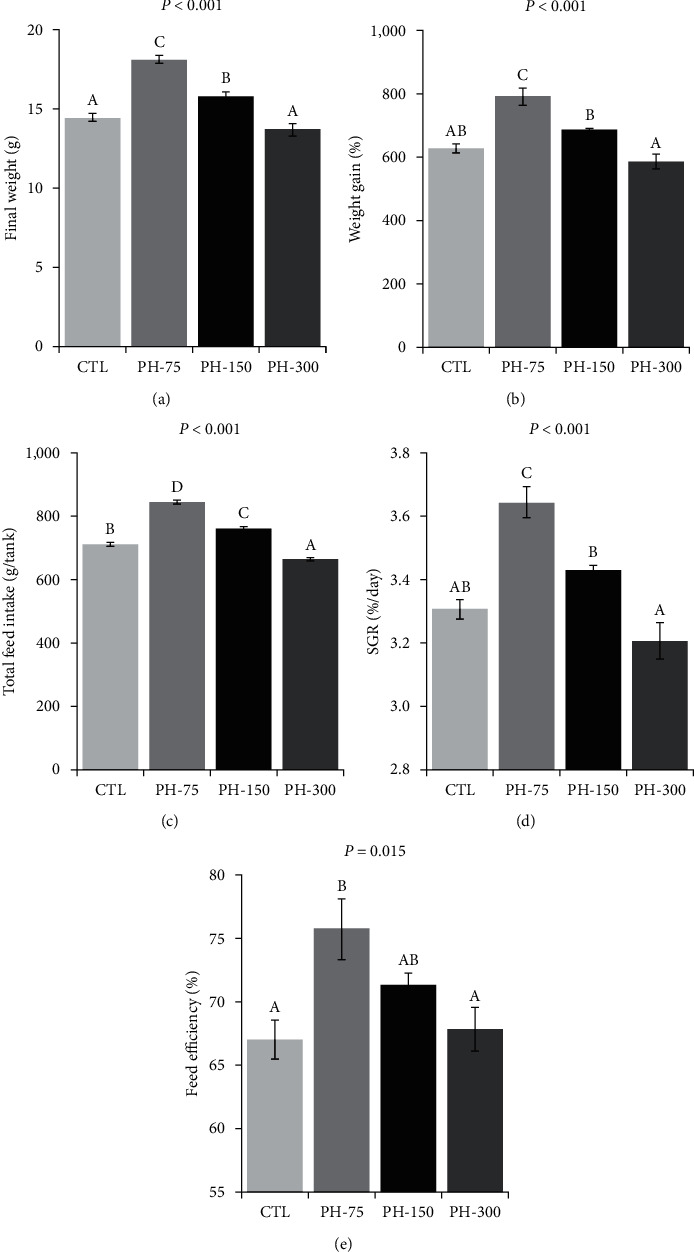
(a–e) Growth performance of Nile tilapia (mean ± standard error) fed diets containing 0, 75, 150, and 300 mg/kg dietary phytol for 60 days. Different letters above the bars show significant differences among the treatments (*n* = 4; Duncan).

The 60-day rearing did not result in any mortality. Dietary phytol had a significant impact on fish growth performance and feed efficiency. As depicted in Figure 1(a)–1(e), the highest final weight and feed intake were observed in the PH-75 treatment. Also, these parameters in the PH-75 and PH-150 treatments were significantly higher than those of the CTL and PH-300 treatments. The feed intake in PH-300 treatment was significantly lower than that of CTL. The weight gain, feed efficiency, and SGR of PH-75 treatment exhibited significant elevations.

**Figure 2:**
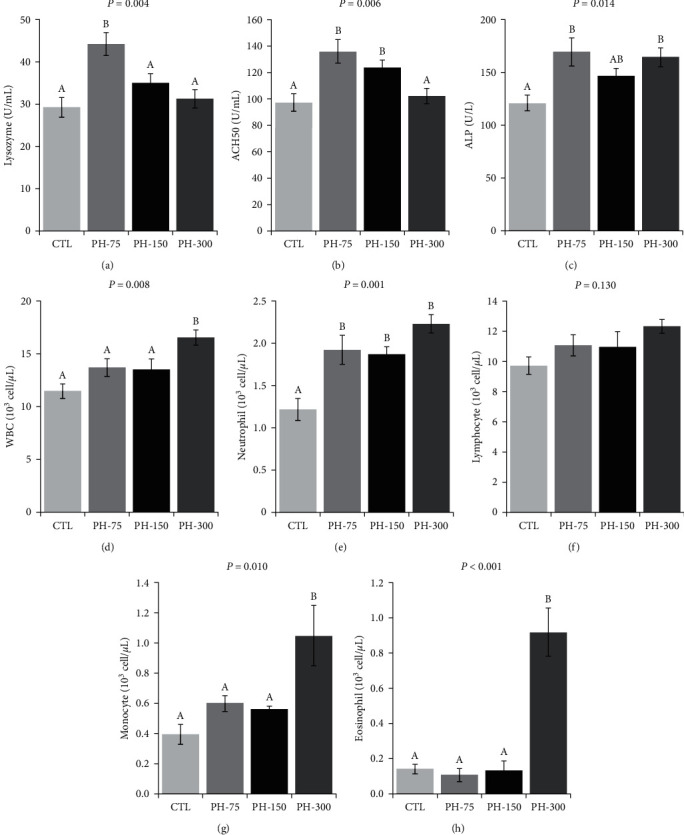
(a–h) Plasma lysozyme, ACH50, and ALP activities and blood WBC counts of Nile tilapia (mean ± standard error) fed diets containing 0, 75, 150, and 300 mg/kg dietary phytol for 60 days. Different letters above the bars show significant differences among the treatments (*n* = 4; Duncan).

The plasma lysozyme activity significantly increased in the PH-75 treatment compared to the CTL treatment (Figure 2(a)). The P-75 and PH-150 had significantly higher plasma ACH50 activities than that of the CTL treatment (Figure 2(b)). The plasma ALP activities significantly increased in the PH-75 and PH-150 treatments compared to the CTL treatment (Figure 2(c)). Phytol treatment induced no significant changes in the blood lymphocyte count (Figure 2(d)), but all phytol-treated fish had significantly higher blood neutrophil count, compared to the CTL (Figure 2(e)). In PH-300 treatment, the blood counts of WBC, monocyte, and eosinophil were significantly higher than the other treatments (Figures 2(d), 2(g), and, 2(h)).

**Figure 3:**
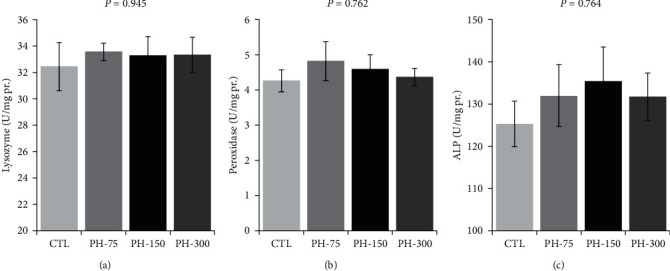
(a–c) Innate immune parameters of skin mucus of Nile tilapia (mean ± standard error) fed diets containing 0, 75, 150, and 300 mg/kg dietary phytol for 60 days. Different letters above the bars show significant differences among the treatments (*n* = 4; Duncan).

There were no significant effects of dietary phytol on the skin mucus lysozyme, peroxidase, and ALP activities (Figure 3(a)–3(c)).

**Figure 4:**
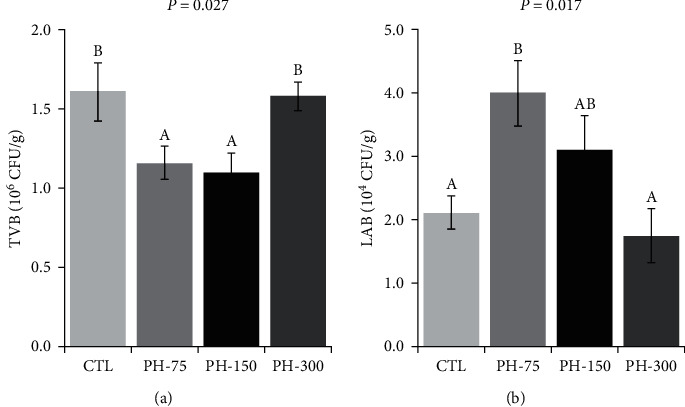
(a, b) Intestinal TVB and LAB of Nile tilapia (mean ± standard error) fed diets containing 0, 75, 150, and 300 mg/kg dietary phytol for 60 days. Different letters above the bars show significant differences among the treatments (*n* = 4; Duncan).

The intestinal TVB (Figure 4(a)) and LAB (Figure 4(b)) counts significantly differed among the treatments. TVB significantly decreased in PH-75 and PH-150, compared to the CTL and PH-300 treatments, while the intestinal LAB count significantly increased in the PH-75 treatment, compared to the CTL.

**Figure 5:**
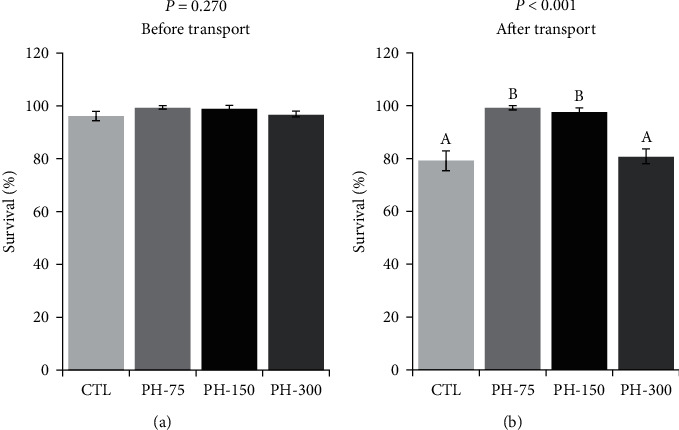
(a, b) Survival of Nile tilapia (mean ± standard error) fed diets containing 0, 75, 150, and 300 mg/kg dietary phytol for 60 days, before and after 6 hr transportation. Different letters above the bars show significant differences among the treatments (*n* = 4; Duncan).

There was no significant difference in the fish survival among the treatments, before transportation (Figure 5(a)). The fish survival after transportation was significantly affected by dietary phytol levels, as the highest survival was related to PH-75 and PH-150 treatments, which were significantly higher than the other treatments (Figure 5(b)).

**Figure 6:**
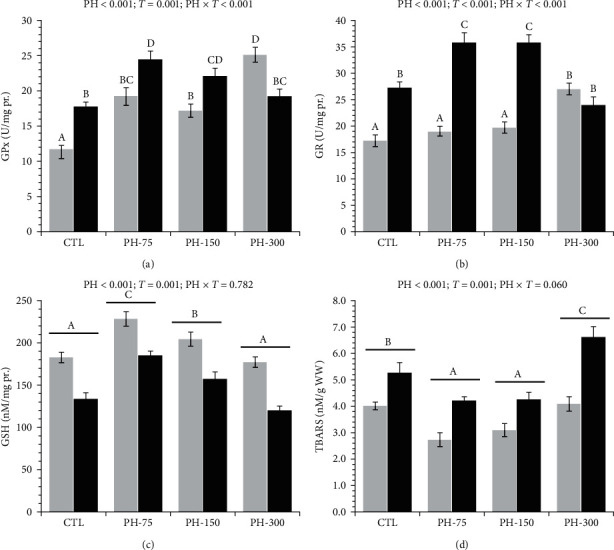
(a–d) Hepatic antioxidant parameters of Nile tilapia (mean ± standard error) fed diets containing 0 (CTL), 75 (PH-75), 150 (PH-150), and 300 (PH-300) mg/kg dietary phytol for 60 days, before and after 6 hr transportation (white bars: before transportation; black bars: after transportation). Different letters above the bars show significant differences among the treatments (*n* = 4; Duncan).

Dietary phytol and transportation had interaction effects on the hepatic GPx (Figure 6(a)) and GR (Figure 6(b)) activities. Before transportation, all phytol-treated fish had significantly higher hepatic GPx activities, compared to the CTL, and the highest activity was related to the PH-300. Transportation led to significant elevations in the hepatic GPx activity in CTL, PH-75, and PH-150 treatments, but a decrease in PH-300 treatment. Prior to the transportation, PH-300 exhibited significantly higher hepatic GR activities in comparison to the other treatments. In CTL, PH-75, and PH-150 treatments, the hepatic GPx activity was significantly elevated, but not in the PH-300 treatment.

Dietary phytol and transportation had significant effects on the hepatic GSH (Figure 6(c)) and TBARS (Figure 6(d)) levels. The concentrations of hepatic GSH significantly increased in the PH-75 and PH-150 treatments, with the highest concentration in the PH-75 treatment. Furthermore, these two treatments exhibited similar levels of hepatic TBARS, which were significantly lower than the levels observed in the other treatments. The highest hepatic TBARS level was observed in PH-300 treatment.

## 4. Discussion

The results indicate that dietary phytol exhibits growth-promoting properties in Nile tilapia, in contrast to those observed in common carp-fed diets containing 125–500 mg/kg phytol [16]. Hence, it appears that Nile tilapia necessitates lower concentrations of dietary phytol for growth promotion, in comparison to common carp. There are no other studies addressing this topic in fish, but dietary supplementation with aquatic plants, which are abundant in phytol, improved fish growth performance. For example, dietary fortification with common duckweed, *Lemna minor* meal [26], *Sargassum oligocystum* hot-water extract [27], and *Entromorpha intestinalis* extract [28] significantly improved the growth performance of rohu, *Labeo rohita*, Iridescent shark, *Pangasius hypophthalmus*, and electric yellow cichlid, *Labidochromis caeruleus*, respectively.

In defending the body against infections, the humoral immune system, carrying WBC and antimicrobial agents, plays critical roles. The effects of diet on fish humoral immune parameters are widely acknowledged, and certain feed additives, such as phytochemicals, have the potential to enhance these parameters [29]. Lysozyme is a natural bactericidal molecule that has the ability to directly kill Gram-positive bacteria by membrane attack and indirectly kill Gram-negative bacteria by stimulating the complement system and phagocytes [30]. Furthermore, the complement proteins have diverse functions in the fish innate and adaptive immune defenses, including opsonization, cell lysis, B cell activation, and inflammation [31]. Lysozyme and complement activities exhibit remarkable elevations during fish infection to eradicate pathogens; however, certain bacteria have the ability to deactivate them [32–35]. Hence, the enhancement of plasma lysozyme and complement activities by dietary phytol may prove advantageous in safeguarding the fish against subsequent infections, as evidenced by previous studies on other feed additives [36, 37]. Similar to the present findings, common carp-fed 125–500 mg/kg dietary phytol exhibited elevations in lysozyme and complement activities [16].

Alkaline phosphatase is an essential enzyme found in lysosomes, serves as an antibacterial agent, and provides protection for fish during wound healing, parasitic infection, and stressful situations. It has an anti-inflammatory role during pathogen invasion as it dephosphrilates inflammation-triggering moieties like bacterial lipopolysaccharides [38]. In fish, neutrophils possess ALP in their lysosomes [39]. Furthermore, studies have demonstrated that dietary feed additives have the potential to enhance circulating ALP activity, subsequently leading to a higher degree of disease resistance [40–42]. Therefore, it is possible that plasma ALP may have originated from neutrophils, at least partially, which would explain the present findings.

Lymphocytes are involved in antigen production and adaptive immune responses, which are typically late but lasting responses. On the contrary, granulocytes are principally involved in the initial phase of immune defense against pathogens. Neutrophils play a significant role in the defense against fish diseases, and their proper functioning is imperative for ensuring the health of fish. They are responsible for recognizing and engulfing invading pathogens, releasing lysozyme and reactive oxygen species to kill them, and recruiting other immune cells to the site of infection [43]. Monocytes play a significant role in the immune system of fish by phagocytosing pathogens, producing inflammatory cytokines, and activating other immune cells [44]. Eosinophils are rare in fish blood, but they are involved in inflammatory responses [45]. The present results suggest that dietary phytol at 75 and 150 mg/kg may improve disease resistance of Nile tilapia, considering the higher blood neutrophil counts. The present results are in agreement with those found in other fish after treatments with different herbal additives such as Hyssop, *Hyssopus officinalis*, extract [46] and neem, *Azadirachta indica*, leaf meal [47]. On the other hand, the increase in the blood monocyte and enosinophil in PH-300 treatment can be signs of inflammation, which is supported by the highest levels of oxidative stress, mortality, and impaired antioxidant responses before/after transportation.

Skin mucosal lysozyme acts as a surface bactericidal agent [48], whereas skin mucosal peroxidase plays a role in the formation of a potent bactericidal and cytotoxic agent known as peroxidase-H_2_O_2_-halide complex [18]. ALP activity in the skin mucus can detoxify proinflammatory compounds generated by microbes [49]. The maintenance of strong skin mucosal immunity is imperative for the prevention of diseases in aquaculture, as water serves as the primary conduit for pathogen transmission. A robust mucosal defense system has the ability to effectively prevent the infiltration of pathogens into the fish body [50]. The present findings indicate that phytol is incapable of inducing skin mucosal immunity, despite activating systemic immunity. However, a bath pathogenic challenge could be better reflected if dietary phytol participates in disease prevention through the stimulation of surface immunity.

The microbes present in the intestine of fish provide a multitude of advantages, including aiding in digestion by metabolizing intricate carbohydrates and proteins and facilitating their absorption [51]. They also play a crucial role in the immune system of fish by producing antimicrobial substances that safeguard the fish from harmful pathogens [52]. Overall, fish gut microbes are crucial for maintaining fish health and well-being, making them a necessary aspect of aquaculture management. The composition of the microbial population in the fish gut is influenced by diet, and phytochemicals and NICs have the potential to influence this structure, as they possess antimicrobial properties [53]. Phytol exhibits bactericidal properties against a diverse range of harmful bacteria, including *Bacillus thermoamylovorans* [54], *Pseudomonas aeruginosa* [55, 56], *Escherichia coli* [57], and *Clostridium sporogenes*, *Sarcina lutea*, *Enterococcus faecalis* [58]. Phytol has the ability to induce oxidative stress, DNA damage, and membrane damage in bacterial cells [55, 59], which explains the mechanism behind its antibacterial effect. A study has demonstrated that phytol inhibits quorum sensing and biofilm formation in *Vibrio campbellii*, an important aquaculture pathogen, both *in vitro* and *in vivo*. Additionally, the tomato clownfish, *Amphiprion frenatus*, demonstrated a higher survival rate after an experimental infection by the bacterium, attributed to lower pathogen localization [60]. In the present study, phytol exhibited a decrease in TVB levels in the intestine of tilapia, thereby demonstrating its antibacterial properties. As evidenced by this study, a reduction in TVB may provide additional resources and space for minor intestinal genera, such as LAB, to facilitate their dominance. Previous studies on this species have demonstrated that an increase in intestinal LAB has been associated with enhanced growth and health of the fish [61–63]. Therefore, it is plausible that phytol may have contributed to the intestinal and systemic health of Nile tilapia in the present study. There is currently no comparable study available for comparison, however, the utilization of dietary herbal additives such as thymol [64], common sage, *Salvia officinalis* [65], and *Gracilaria gracilis* extract [66], has had a significant impact on the diversity and structure of intestinal bacterial populations in fish.

Glutathione-related antioxidant enzymes, along with direct radical-scavenging capacity of GSH, safeguard cells against the harmful effects of oxidative stress caused by excessive reactive oxygen species [67, 68]. These antioxidants are often elevated after stressful events; stress triggers an increase in cell respiration and the production of superoxide anions, which require detoxification by superoxide dismutase [69]. The resulting hydrogen peroxide undergoes detoxification by GPx by utilizing GSH as a cofactor, resulting in GSH oxidation [67]. Subsequently, GR transforms the oxidized GSH into its reduced form [70]. During transportation, fish may encounter various stressors, including elevated oxygen levels, fluctuations in water quality, and accumulation of water ammonia [71]; all of which can result in an increase in the production of reactive oxygen species. If the antioxidant defense system of the fish is overwhelmed, oxidative damage may occur, resulting in cellular and tissue damage, compromised immune function, and elevated susceptibility to disease [72, 73]. The present findings indicate that the mortality of the fish exhibited a pattern akin to that of lipid peroxidation, indicating that oxidative stress may be a potential cause of mortality. Phytol has been demonstrated to possess antioxidant properties [74]; a study on common carp has shown that dietary phytol can induce hepatic antioxidant enzymes and mitigate ammonia-induced lipid peroxidation [16]. It has been demonstrated that the incorporation of antioxidant phytochemicals, such as turmeric [10], or other antioxidants, such as ascorbic acid [75] and glycine [76], into the diet of fish can enhance antioxidant capacity and mitigate post-transportation oxidative stress. The antioxidant responses before and after transportation indicate that 75 and 150 mg/kg phytol can support the antioxidant system and suppress lipid peroxidation. Although dietary 300 mg/kg phytol was harmful to Nile tilapia, it suppressed the GPx and GR activities, thereby increasing lipid peroxidation.

## 5. Conclusion

Phytol can be a suitable feed additive for Nile tilapia, which increases growth performance, humoral immune parameters, and intestinal beneficial bacteria. Furthermore, it acts as an antioxidant agent, reducing post-transportation oxidative stress and mortality. As per the present findings, it has been determined that a dosage of 75 mg/kg phytol is suitable for Nile tilapia, whereas a dosage of 300 mg/kg should be avoided as it may result in an increase in lipid peroxidation.

## Figures and Tables

**Table 1:** Dietary ingredients and proximate composition.

Ingredients (g/kg)	Dietary phytol levels (mg/kg)			
	0 (CTL)	75 (PH-75)	150 (PH-150)	300 (PH-300)
Corn meal^a^	123	123	123	123
Wheat flour^b^	241	241	241	241
Soybean oilcake^c^	306	306	306	306
Poultry by-product^d^	252	252	252	252
Fish canning by-product^e^	30	30	30	30
Plant oil (corn oil + sunflower oil; 1 : 1 ratio)	21	21	21	21
Vitamin + mineral premix^f^	15.5	15.5	15.5	15.5
Methionine^g^	3	3	3	3
Lysine^h^	3	3	3	3
Salt	5	5	5	5
Bentonite^i^	0.5	0.425	0.35	0.2
Phytol^j^	0	0.075	0.15	0.3
Proximate composition (g/kg)				
Moisture	88.6	88.3	88.3	88.7
Crude protein	345	348	341	344
Crude fat	87.5	86.4	88.1	86.7
Crude ash	69.1	69.0	68.2	68.0
Crude fiber	37.1	37.1	38.0	36.8

^a^Containing 8.9%, 3.5%, 5.3%, and 2.6% of crude protein, fat, ash, and fiber, respectively. ^b^Containing 11.1%, 1.5%, 2.3%, and 2.5% of crude protein, fat, ash, and fiber, respectively. ^c^Containing 44.3%, 1.88%, 5.32%, and 3.68% of crudeprotein, fat, ash, and fiber, respectively. ^d^Containing 63%, 16%, 10%, and 4% of crude protein, fat, ash, and fiber, respectively. ^e^Containing 56%, 18%, 4%, and 2% of crude protein, fat, ash, and fiber, respectively. ^f^Supplied by Amineh Gostar Co., Tehran, Iran, providing per kg if diet: B2: 10 mg; E: 20 mg; K: 24 mg; B3: 12 mg; B5: 40 mg; B6: 5 mg; B1: 4 mg; A: 1,600 IU; D3: 500 IU; H: 0.2 mg; B9: 2 mg; B12: 0.01 mg; C: 60 mg; Inositol: 50 mg; Iodate: 0.05 mg; Fe: 2.5 mg; Co: 0.04 mg; Cu: 0.5 mg; Zn: 6 mg; Choline: 150 mg; Se: 0.15 mg; Mn: 5 mg. ^g^CJ CheilJedang Corporation, Seoul, South Korea. ^h^Domisha Industrial Production Co., Tehran, Iran. ^i^Ninety nine percent purity; Sigma–Aldrich Co., Saint Louise, USA.

## Data Availability

Data are available upon a reasonable request from the corresponding author.
